# Delineation of a frequency-organized region isolated from the mouse primary auditory cortex

**DOI:** 10.1152/jn.00932.2014

**Published:** 2015-02-18

**Authors:** Hiroaki Tsukano, Masao Horie, Takeshi Bo, Arikuni Uchimura, Ryuichi Hishida, Masaharu Kudoh, Kuniyuki Takahashi, Hirohide Takebayashi, Katsuei Shibuki

**Affiliations:** ^1^Department of Neurophysiology, Brain Research Institute, Niigata University, Niigata, Japan;; ^2^Division of Neurobiology and Anatomy, Graduate School of Medicine and Dental Sciences, Niigata University, Niigata, Japan;; ^3^KOKORO-Biology Group, Laboratories for Integrated Biology, Graduate School of Frontier Biosciences, Osaka University, Osaka, Japan;; ^4^Department of Physiology, Teikyo University School of Medicine, Tokyo, Japan; and; ^5^Department of Otolaryngology, Graduate School of Medicine and Dental Sciences, Niigata University, Niigata, Japan

**Keywords:** auditory cortex, mapping, multiple frequency organization, courtship song, mice

## Abstract

The primary auditory cortex (AI) is the representative recipient of information from the ears in the mammalian cortex. However, the delineation of the AI is still controversial in a mouse. Recently, it was reported, using optical imaging, that two distinct areas of the AI, located ventrally and dorsally, are activated by high-frequency tones, whereas only one area is activated by low-frequency tones. Here, we show that the dorsal high-frequency area is an independent region that is separated from the rest of the AI. We could visualize the two distinct high-frequency areas using flavoprotein fluorescence imaging, as reported previously. SMI-32 immunolabeling revealed that the dorsal region had a different cytoarchitectural pattern from the rest of the AI. Specifically, the ratio of SMI-32-positive pyramidal neurons to nonpyramidal neurons was larger in the dorsal high-frequency area than the rest of the AI. We named this new region the dorsomedial field (DM). Retrograde tracing showed that neurons projecting to the DM were localized in the rostral part of the ventral division of the medial geniculate body with a distinct frequency organization, where few neurons projected to the AI. Furthermore, the responses of the DM to ultrasonic courtship songs presented by males were significantly greater in females than in males; in contrast, there was no sex difference in response to artificial pure tones. Our findings offer a basic outline on the processing of ultrasonic vocal information on the basis of the precisely subdivided, multiple frequency-organized auditory cortex map in mice.

the primary sensory cortex of the mammalian brain is widely known to receive the first thalamic inputs that convey peripheral sensory information, such as hearing and vision. In the auditory cortex, the primary auditory cortex (AI) is the main recipient of information from the ears into the cortex and transfers this information to higher-order auditory cortical regions (Kaas and Hackett 2000). The AI is important in terms of being both the receiver and the relay point in hierarchical auditory processing and has been studied using various animals, including mice.

The mouse is widely used in auditory physiological research because of its merits as an animal model in auditory cortical studies involving two-photon Ca^2+^ imaging (Bandyopadhyay et al. 2010; Bathellier et al. 2012; Honma et al. 2013; Issa et al. 2014; Rothschild et al. 2010), voltage-sensitive imaging (Sawatari et al. 2011; Takahashi et al. 2006), anatomical studies (Barkat et al. 2011; Hackett et al. 2011; Hofstetter and Ehret 1992; Horie et al. 2013; Llano and Sherman 2008; Oviedo et al. 2010), genetic manipulation (Barkat et al. 2011; Rotschafer and Razak 2013; Xiong et al. 2012), and behavioral analysis (Tsukano et al. 2011). Accordingly, the attainment of precise knowledge of the AI in mice is essential. However, the delineation of the map of the mouse auditory cortex is under debate, as maps obtained by electrophysiology have been amended in recent studies using optical imaging.

The classical mouse auditory cortex map was drawn using unit recording. The anterior auditory field (AAF) and AI with clear frequency gradients are placed at the center as the core and are surrounded by the belt region, considered the higher-order region, including the secondary auditory field (AII), the ultrasonic field (UF), and the dorsoposterior field (DP) (Stiebler et al. 1997). The AAF and AI have frequency-organized arrangements covering the frequencies up to 40 kHz; neurons with characteristic frequency over 50 kHz are localized in the UF (Fig. 1*A*). A subsequent study indicated that a distinct UF region does not exist, with the AAF and AI having the full range of frequency organization, processing sounds from 4 kHz up to 64 kHz (Guo et al. 2012). The term “UF” has thus been redefined as subparts within the AAF and AI that process high-frequency sounds (Fig. 1*B*). Recently, however, Issa et al. (2014) have reported elegantly that the AI is divided into two rostral areas that process high-frequency tones, despite only one caudal area being activated by low-frequency tones. This leads to the generation of forked, dual-frequency gradients inside of the AI (Issa et al. 2014). The larger dorsal branch of the fork-shaped frequency gradients travels toward the high-frequency area of the AI, referred to as the UF, as reported by Guo et al. (2012), whereas the smaller ventral division has an axis of the frequency organization toward the AII (Fig. 1*C*) (Issa et al. 2014). This new map was obtained using optical imaging. Moreover, they clearly revealed that the direction of the frequency organization of the AAF was present and is directed from the dorsorostral to ventrocaudal direction. Their study used transgenic mice, in which the calcium-sensitive protein GCaMP3 was initially expressed uniformly, offering much better spatial resolution regarding the auditory cortical surface than that achieved by unit recording. Principally, however, previous anatomical studies reported that frequency-organized maps in the AAF and AI reflect distinct topological projections from frequency-organized maps in the medial and lateral parts, respectively, of the ventral division of the medial geniculate body (MGv; MGB) in mice as well (Horie et al. 2013; Takemoto et al. 2014). Moreover, various mammals have multiple frequency-organized regions with distinct, unique frequency direction (Higgins et al. 2010; Kalatsky et al. 2005; Kanold et al. 2014); spectral, temporal, and spatial sensitivities (Bizley et al. 2009; Imaizumi et al. 2004; Polley et al. 2007); and thalamocortical projections (Lee et al. 2004; Storace et al. 2010, 2012) without redundancy. The mouse AI, therefore, would be exceptional if the fork-shaped frequency gradients existed inside of a single region.

**Fig. 1.**
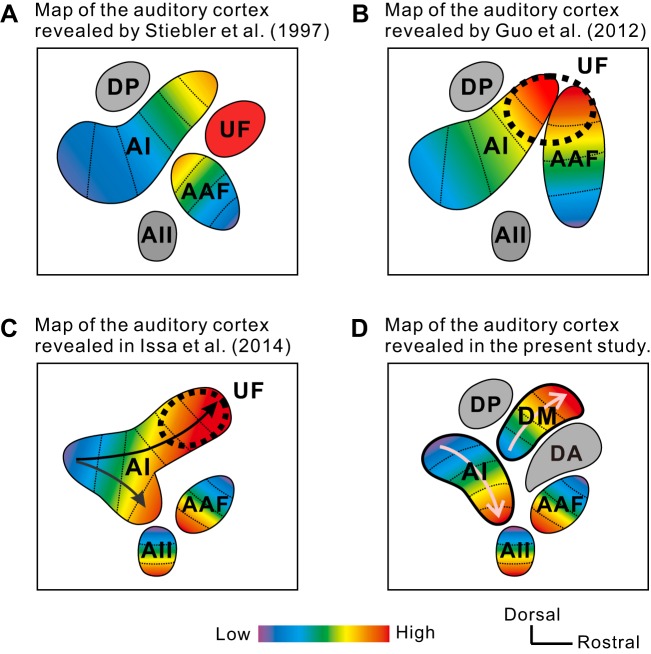
Schematic maps of the mouse auditory cortex in previous studies and the present study. *A*: schematic map of the auditory cortex in Stiebler et al. (1997). Frequency gradients of the anterior auditory field (AAF) and primary auditory cortex (AI) included neurons with characteristic frequencies up to 40 kHz, and neurons with characteristic frequencies higher than 50 kHz are located in the distinct ultrasonic field (UF). *B*: map of the auditory cortex in Guo et al. (2012). The AAF and AI had ultrasonic bands higher than 50 kHz. The UF was interpreted to be high-frequency subparts in the high-frequency area of the AAF and AI but not a distinct region. *A* and *B*: these maps were drawn according to the results obtained by unit recording. *C*: map of the auditory cortex in Issa et al. (2014), showing 2 streams of frequency gradients inside of the AI. The major gradient runs toward the dorsal part, including the UF area, whereas the minor gradient runs toward the secondary auditory field (AII). The direction of the frequency organization of the AAF is drawn along the ventrocaudal axis, consistent with the data reported by Horie et al. (2013). *D*: map of the auditory cortex reported in the present study. Newly delineated, distinct region dorsomedial field (DM) was isolated from the AI high-frequency area. The direction of the frequency organization of the AI is the one traveling toward AII. The direction of the frequency organization of the AAF matches that reported in the previous studies (Horie et al. 2013; Issa et al. 2014). The region that Stiebler et al. (1997) defined as UF [referred to as dorsoanterior field (DA)] responds to slow-frequency modulation (FM) components, regardless of tonal frequency range. *C* and *D*: these maps were drawn according to the results obtained by optical imaging. DP, dorsoposterior field.

In this study, we tried to clarify whether the frequency gradient inside of the AI proceeds in a straight line or shows a forked shape. For this purpose, we used a combination of flavoprotein autofluorescence imaging and anatomical techniques. Flavoproteins are endogenous fluorescent proteins in mitochondria, and fluorescence imaging of these proteins has been used to map precisely the auditory cortex (Honma et al. 2013; Horie et al. 2013; Kubota et al. 2008; Ohshima et al. 2010; Takahashi et al. 2006), visual cortex (Andermann et al. 2011; Tohmi et al. 2009, 2014; Yoshitake et al. 2013), somatosensory cortex (Komagata et al. 2011), and insular cortex (Gogolla et al. 2014). With the use of this technique, we reproduced the finding that the mouse AI is divided into two areas in response to high-frequency tones (Issa et al. 2014; Tsukano et al. 2013a). Additionally, we used SMI-32 immunolabeling, which has been used to partition various cortical regions (Boire et al. 2005; Budinger et al. 2000; Mellott et al. 2010; Ouda et al. 2012; Paxinos et al. 2009; Rothschild et al. 2010; van der Gucht et al 2001; Wong and Kaas 2009) to investigate the cytoarchitecture in the two AI high-frequency areas. We found that the dorsal part of the AI high-frequency area is a different region from the rest of the AI, including the low-frequency area and the ventral high-frequency area, and the posterior frequency-organized region, which is referred to as the AI, is restricted to the low-frequency area and the ventral high-frequency area that has been considered as a supplemental AI area in the study by Issa et al. (2014) (Fig. 1*D*). Existence of multiple frequency organizations in the mouse auditory cortex may unify the auditory cortical maps of mice and other mammals.

## METHODS

### 

#### Animals.

The Committee for Animal Care at Niigata University approved the experimental protocols used in this study. We used 5- to 7-wk-old C57BL/6 mice (*n* = 197; Charles River Japan, Yokohama, Japan), 7- to 9-wk-old Balb/c mice (*n* = 3; Charles River Japan), and 7- to 9-wk-old CBA/CaJ mice (*n* = 3; The Jackson Laboratory, Bar Harbor, ME). The animals were housed in cages with ad libitum access to food pellets and water and were kept on a 12-h light/dark cycle. Male and female mice used in the experiments (see Fig. 14) were 6-wk-old C57BL/6 mice, produced in our institute, and they were not housed with mice of the opposite sex after weaning at 3 wk old.

#### In vivo flavoprotein fluorescence imaging.

**Fig. 2.**
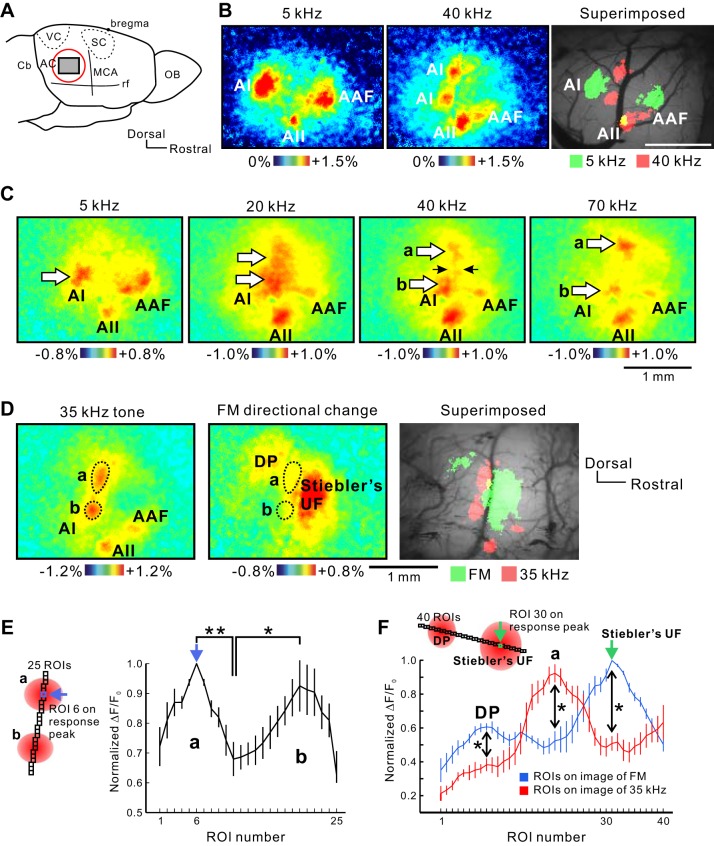
Two distinct responses to high-frequency tones in the AI. *A*: schematic drawing of the mouse auditory cortex. Auditory cortex (AC); cerebellum (Cb); medial cerebral artery (MCA); olfactory bulb (OB); rhinal fissure (rf); somatosensory cortex (SC); visual cortex (VC). *B*: neural responses to a 5- or 40-kHz tone obtained by flavoprotein fluorescence imaging in a mouse with craniotomy. The AI in response to a 40-kHz tone area was divided into 2 parts. Three images were obtained from the same mouse. Scale bar, 1 mm. *C*: neural responses to 5–70 kHz tones revealed by transcranial flavoprotein fluorescence imaging. The caudal AI low-frequency area was clearly activated. When 20–70 kHz tones were presented, the AI high-frequency area was divided into 2 parts: the dorsal part (a) and the ventral part (b), indicated by white arrows. Four images were obtained from the same mouse. *D*: positional relationship between the dorsal part of the AI high-frequency area and Stiebler's UF and DP. Stiebler's UF and DP were activated by slow FM directional changes (Honma et al. 2013). Three images were obtained from the same mouse. *E*: quantitative analysis of response slopes by a 35-kHz tone. Regions of interest (ROIs) were placed across the 2 highest peaks in the AI high-frequency areas, and the significant double peaks in fluorescence change (ΔF)/baseline intensity (F_0_) were drawn (*P < 0.05; **P < 0.01; Wilcoxon signed-rank test, 10 mice). Each value was normalized as percent of the peak value at ROI 6, which was put on the response peak of the dorsal AI high-frequency area and used as a landmark to register across individuals. Sites a and b are equivalent to those shown in *C*. *F*: quantitative analysis of response slopes on the response in the experiment shown in *D*. ROIs were placed across the peaks in Stiebler's UF and DP, and the same ROIs were placed onto the image of the responses to a 35-kHz tone obtained from the same mouse (*inset*). The red line was obtained from the response to a 35-kHz tone, and the blue line was obtained from the response to FM directional changes in the same mouse. The response peak derived from the dorsal AI high-frequency area was placed in the middle of Stiebler's UF and DP (*P < 0.05; Wilcoxon signed-rank test, 6 mice). Each value was normalized as percent of the peak value at ROI 30, which was put on the response peak of Stiebler's UF and used as a landmark to register across individuals. Transcranial imaging was performed in experiments *C–F*.

**Fig. 3.**
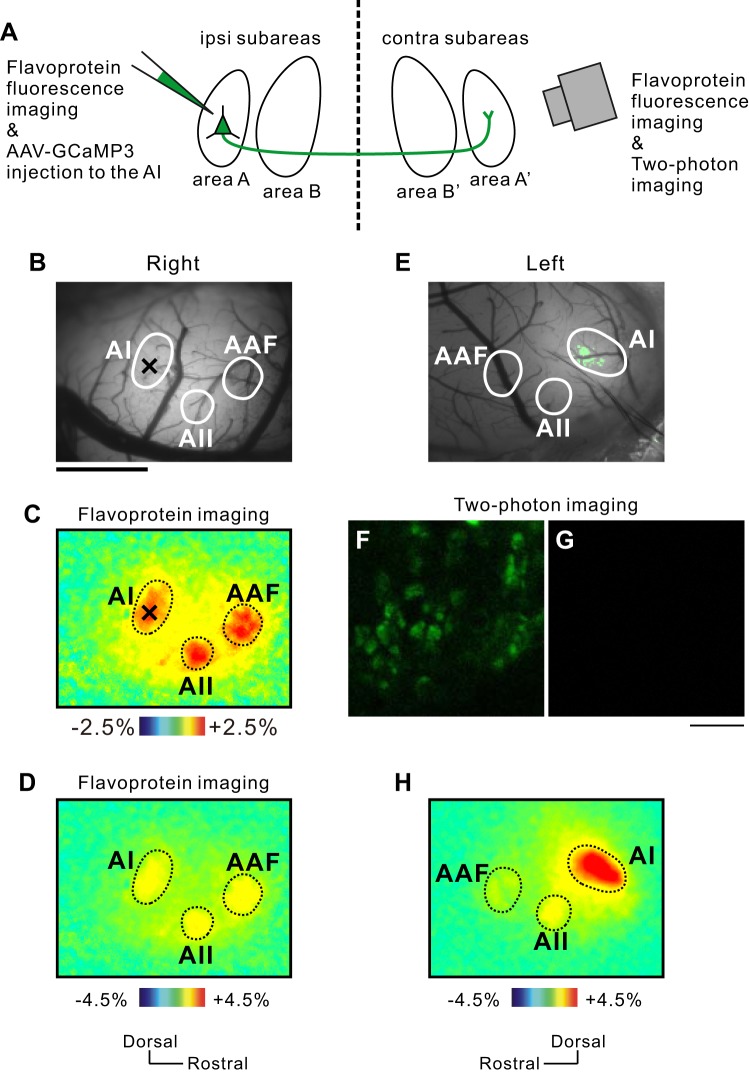
Interhemispheric projection between the auditory cortices. *A*: schematic drawing showing that a neuron in the subarea in the auditory cortex projects to the equivalent contralateral subarea. *B–D*: injection site of adeno-associated virus (AAV)-GCaMP3 in the right auditory cortex. The areal borders were drawn according to the flavoprotein fluorescence response to a 5-kHz tone (*C*). *C* and *D*: the images shown are the same except for the color range. ×, injection site. *E*: GCaMP3-positive axon terminals in the AI 5-kHz area in the left auditory cortex observed using a stereoscopic microscope. The top 10% pixels in intensity are colored green. *F*: GCaMP3-positive axon terminals visualized using a 2-photon microscopy in the place where an intense signal was observed in *E*. *G*: no GCaMP3-positive terminals found outside of the AI 5-kHz area. *H*: the large calcium responses to a 5-kHz tone derived from GCaMP3-positive terminals in the left AI. This response was obtained using an epifluorescent microscope under the same experimental condition as that to perform usual flavoprotein fluorescence imaging. Only the AI showed a much larger response in *H* due to GCaMP-stained terminals. Flavoprotein fluorescence responses, which were small compared with GCaMP signals, were observed in the 5-kHz area in the AAF and AII. Scale bars, 1 mm (*B*); 10 μm (*F* and *G*).

**Fig. 4.**
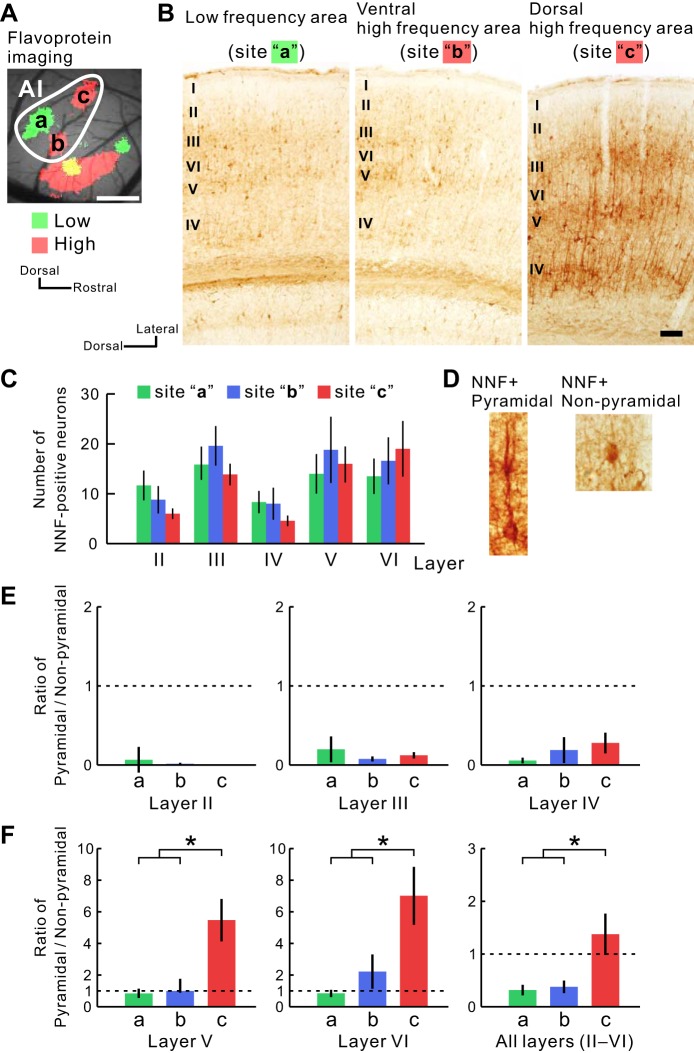
Histological properties of the new region different from the AI in SMI-32 immunolabeling. *A*: functional location of the AI obtained using flavoprotein fluorescence imaging. Scale bar, 500 μm. *B*: sample images of SMI-32 immunolabeling patterns. We observed nonphosphorylated neurofilament (NNF) at sites a, b, and c, indicated in *A*. Scale bar, 100 μm. *C*: number of NNF-positive neurons in each area of the AI. Layers were identified based on Nissl staining in an adjacent section. *D*: typical images of an NNF-positive pyramidal and nonpyramidal neuron. *E*: ratio of the number of NNF-positive pyramidal/nonpyramidal neurons in the superficial layers (II, III, and IV). *F*: ratio of the number of NNF-positive pyramidal/nonpyramidal neurons in the deep layers (V and VI) and all of the layers (II–VI). *P < 0.05, Tukey-Kramer's test. Site a, *n* = 6; site b, *n* = 6; site c, *n* = 7. NNF and biotinylated dextran amine (BDA) were visualized in the adjacent tissue sections.

**Fig. 5.**
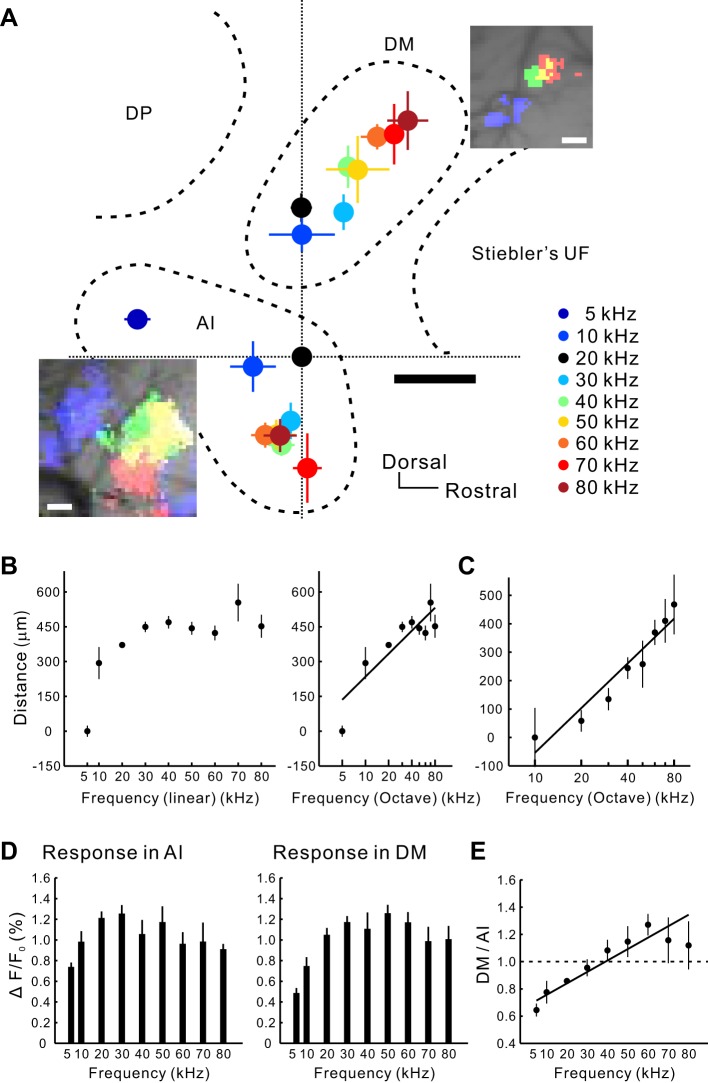
Frequency organizations of the AI and DM revealed by flavoprotein fluorescence imaging. *A*: 2 frequency gradients for 5–80 kHz sounds in the AI and newly identified DM revealed by flavoprotein fluorescence imaging. The location of the 20-kHz response peak in the AI was used as the origin of coordinates on which data from several animals were superimposed. Black scale bar, 200 μm. See color-coding key on right. *Top inset*: response peak shifts in DM. Blue, 40 kHz; green, 60 kHz; red, 80 kHz; yellow, overlap between 60 and 80 kHz. White scale bar, 100 μm. *Bottom inset*: blue, 5 kHz; green, 10 kHz; yellow, 20 kHz; red, 50 kHz. White scale bar, 100 μm. *B*: a frequency gradient of the linear (*left*) and the log scales (*right*) in the AI. The horizontal axis is a line connecting points of 5 and 80 kHz (*r* = 0.85; *P* < 0.001, Spearman's rank correlation test). The slope in the octave axis was 1.01 octave/125 μm. *C*: a frequency gradient in the DM (*r* = 0.69; *P* < 0.001, Spearman's rank correlation test). The slope was 1.17 octave/125 μm. *D*: response amplitudes against each frequency in the AI (*left*) and DM (*right*). *E*: ratio of amplitudes of the DM to AI by each stimulus frequency (*r* = 0.63, *P* < 0.001, Spearman's rank correlation test). 5 kHz, *n* = 15; 10 kHz, *n* = 5; 20 kHz, *n* = 15; 30 kHz, *n* = 8; 40 kHz, *n* = 6; 50 kHz, *n* = 7; 60 kHz, *n* = 8; 70 kHz, *n* = 3; 80 kHz, *n* = 3.

**Fig. 6.**
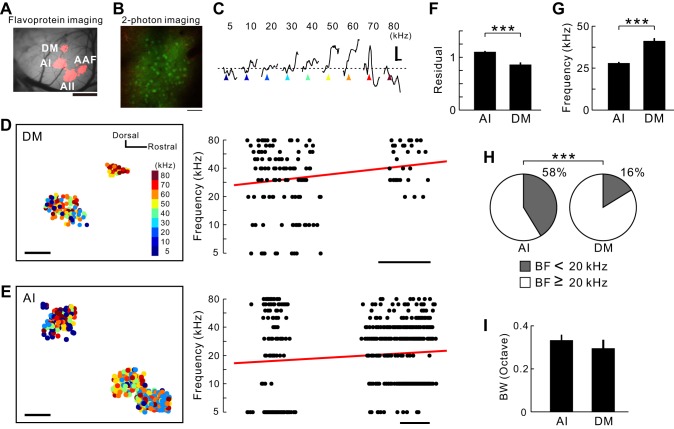
Frequency organizations of the AI and DM revealed by 2-photon imaging. *A*: identification of subregions in the auditory cortex using flavoprotein fluorescence imaging. The image was obtained using a 35-kHz tone. Scale bar, 1 mm. *B*: example of staining with Fura-2 and sulforhodamine 101 in the auditory cortex. Scale bar, 50 μm. *C*: sample traces of calcium response in a neuron with a best frequency (BF) of 60 kHz. Horizontal scale, 3 s; vertical scale, 2%; triangles, timing when a tone was given. A series of sounds at 80 dB sound pressure level was presented to a mouse. *D*: distribution of neurons in the DM. Data were obtained from 3 animals, and images were superimposed on the location of a 5-kHz response peak in the AI as the origin of coordinates obtained using flavoprotein fluorescence imaging. Color of each plot represents its BF. A significant, progressive increase in frequency was observed (*P* < 0.05, Spearman's rank correlation test; 163 neurons). Scale bar, 200 μm. *E*: distribution of neurons in the AI. Data were obtained from 4 animals and reconstructed. A significant, progressive increase in frequency was observed (*P* < 0.0001, Spearman's rank correlation test; 598 neurons). Scale bar, 200 μm. *F*: comparison of the degree of the frequency organization between the AI and DM. The extent of disorder was evaluated using the distances of each neuron from the regression line (***P < 0.001, Mann-Whitney U-test). *G*: averages of BFs (***P < 0.001, Mann-Whitney U-test). *H*: percentages of neurons with BF ≤ 10 kHz (***P < 0.0001, χ^2^ test). *I*: comparison of neuron bandwidths (BW) between the AI and DM, defined as the logarithmic ratio of minimum and maximum frequencies that resulted in a response >75% (*P* > 0.2, Mann-Whitney U-test) of the peak amplitude.

**Fig. 7.**
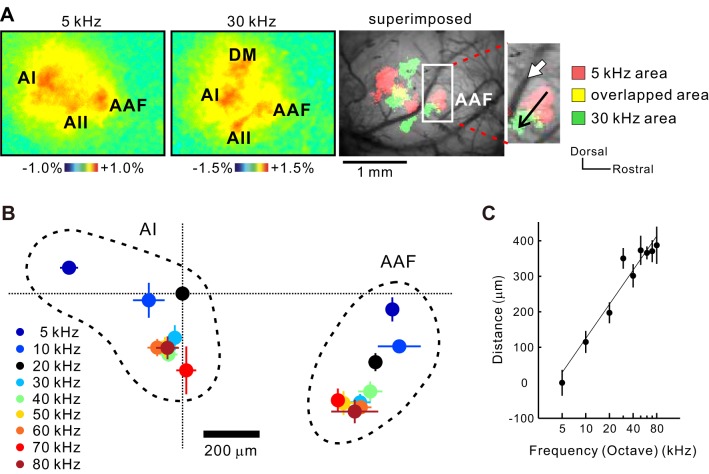
Frequency organization of the AAF and AI. *A*: images of responses to a 5 (*left*)- and 30 (*middle*)-kHz tone and a superimposed image of these 2 images (*right*). The white arrow indicates the region defined as the high-frequency area of AAF in Guo et al. (2012). *B*: frequency-organized maps of the AI and AAF to 5–80 kHz sounds revealed by flavoprotein fluorescence imaging. The response peak of AI to 20 kHz is used as the origin of coordinates to superimpose the data in several animals. *C*: frequency gradient of the AAF. The horizontal axis is a line connecting points of 5 and 80 kHz (5 kHz, *n* = 15; 10 kHz, *n* = 5; 20 kHz, *n* = 15; 30 kHz, *n* = 8; 40 kHz, *n* = 6; 50 kHz, *n* = 7; 60 kHz, *n* = 8; 70 kHz, *n* = 3; 80 kHz, *n* = 3; *P* < 0.001).

**Fig. 8.**
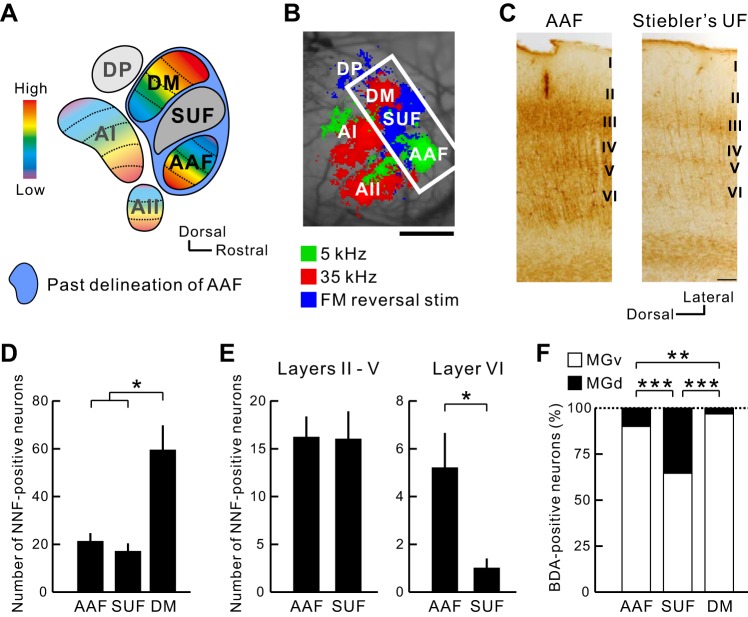
Difference in histological and anatomical properties among the AAF, Stiebler's UF, and DM. *A*: schematic drawing of the auditory cortical map. The area enclosed by the blue oval was the definition of the AAF proposed by Guo et al. (2012). SUF, Stiebler's UF. *B*: auditory cortical map revealed by flavoprotein fluorescence responses. The area enclosed by the white rectangle is equivalent to that in the blue oval in *A*. Scale bar, 500 μm. *C*: sample images of SMI-32 immunolabeling patterns in the AAF and Stiebler's UF. Scale bar, 100 μm. *D*: number of NNF-positive neurons in the AAF, Stiebler's UF, and DM. *P < 0.05, Tukey-Kramer's test; AAF, *n* = 5; Stiebler's UF, *n* = 4; DM, *n* = 7. *E*: number of NNF-positive neurons in the *layer VI* and the others of the AAF and Stiebler's UF. *P < 0.05, Mann-Whitney U-test; AAF, *n* = 5; Stiebler's UF, *n* = 4. *F*: percentage of neuronal distribution in the medial geniculate body [MGB; the ventral division of MGB (MGv) or the dorsal division of MGB (MGd)] by their cortical target. **P < 0.01; ***P < 0.000001, χ^2^ test after Bonferroni correction; to AAF, *n* = 150 neurons from 3 mice; to Stiebler's UF, *n* = 144 neurons from 6 mice; to DM, *n* = 316 neurons from 7 mice.

In vivo flavoprotein fluorescence imaging was performed, as described in our previous studies (Takahashi et al. 2006; Tsukano et al. 2013b). Mice were deeply anesthetized with urethane (1.7 g/kg ip; Wako, Osaka, Japan), and their rectal temperatures were maintained at 37°C. After local anesthesia using bupivacaine, the skin and temporal muscle over the right auditory cortex were incised. A piece of metal was attached to the skull with dental resin, and the head was fixed by screwing the metal piece onto a manipulator. The skull over the auditory cortex was removed in mice used for some experiments (see Figs. 2*B*, 3, 4, 6, 8, 12, and 13) to compare response patterns of the auditory cortex or further tracer-injection experiments. Transcranial imaging was performed in the other experiments. The right auditory cortex was observed unless otherwise noted. The exposed surface of the intact skull was covered with liquid paraffin (Wako) to keep the skull transparent in transcranial imaging. Cortical images (128 × 168 pixels after binning) of endogenous green fluorescence (λ = 500–550 nm) in blue light (λ = 470–490 nm) were recorded using a cooled charge-coupled device (CCD) camera system (AQUACOSMOS with ORCA-R2 camera; Hamamatsu Photonics, Hamamatsu, Japan). Images were taken at 9.7 Hz [54 Hz in some experiments (see Figs. 11 and 14, *C–E*)]. Images were averaged over 20 trials unless otherwise noted. Spatial averaging of 5 × 5 pixels was applied. Images were calculated as fluorescence change (ΔF)/baseline intensity (F_0_), where ΔF = F − F_0. _The F_0_ was obtained by averaging the intensity values during the prestimulus period (∼500 ms). The response amplitude was evaluated as ΔF/F_0_ in a circle window with a diameter of 20 pixels. When the frequency organization was evaluated (see Figs. 5 and 7), a circle window with a diameter of 15 pixels was chosen to give the largest response amplitude, and the location of the center pixel was considered to be the frequency-specific response peak.

#### In vivo two-photon calcium imaging.

Calcium imaging was performed using a two-photon microscope (TCS SP5 MP; Leica Microsystems, Wetzlar, Germany) with a hybrid detector (HyD; Leica Microsystems) and a Ti-Sapphire mode-locked femto second laser (Chameleon Vision; Coherent, Santa Clara, CA), as described in our previous studies (Honma et al. 2013; Tohmi et al. 2014; Yoshitake et al. 2013). Calcium-sensitive dye was prepared by dissolving Fura-2 AM (Invitrogen Life Technologies, Boston, MA) in 20% (w/v) Pluronic F-127 in DMSO (Invitrogen Life Technologies) and diluted with Ringer solution containing sulforhodamine 101 (SR-101; Invitrogen Life Technologies). After anesthetic induction with urethane (1.9 g/kg ip), craniotomy, and localization using flavoprotein fluorescence imaging, a solution of calcium-sensitive dye was pressure injected (5–20 kPa) for 5–10 min into *layer II/III* using glass pipettes (tip diameter: 2–4 μm). Astrocytes were distinguished from neurons using SR-101. After injection, the pipette was withdrawn, and the craniotomy was covered with 2% agarose (1-B; Sigma-Aldrich, St. Louis, MO) and a thin cover glass (thickness <0.15 mm; Matsunami, Osaka, Japan), which was fixed to the skull with dental cement (Sun Medical, Shiga, Japan). Excitation wavelength for Fura-2 was 800 nm, and that for SR-101 was 900–950 nm. Images (256 × 256 pixels) were recorded at 3.7 Hz in a 260 × 260-μm region.

The data were realigned using AQUACOSMOS and MATLAB software (MathWorks, Natick, MA). Data from five to six trials of the same stimulation were averaged. Size-matched regions of interest (ROIs) were chosen. Data were calculated as ΔF/F_0_, where ΔF = F_0_ − F. The F_0_ was obtained by averaging the intensity values during the prestimulus period (∼3 s). The response of each neuron was defined as the maximum value in poststimulus observation windows (∼5 s). Neurons were defined as responsive when the response peak was >4 SD above the baselines during the prestimulus period to minimize errors by baseline fluctuations. The best frequency (BF) of a neuron was defined as the frequency to which the neuron had the greatest response. We examined the correlation between frequency and location and computed a regression line. The direction of the frequency organization was defined so as to make the correlation coefficient as large as possible. Then, we examined the residuals as a measure of deviation from the frequency organization. The bandwidth of tuning curves was defined as the logarithmic ratio of minimum and maximum frequencies that resulted in a response >75% of the peak amplitude.

#### Auditory stimuli.

Tones were made by a computer using a custom-written LabVIEW program (National Instruments, Austin, TX) at a sampling rate of 500 kHz. Courtship songs of a male mouse, emitted when he was placed with a familiar female mouse in estrous in the same cage, were recorded using the recording software Avisoft-RECORDER (Avisoft Bioacoustics, Glienicke, Germany) at a sampling frequency of 250 kHz, with a microphone (CM16/CMPA; Avisoft Bioacoustics) and a preamplifier (UltraSoundGate 116; Avisoft Bioacoustics). Sounds were low-pass filtered at 150 kHz (3624; NF, Kanagawa, Japan). Pure tones at frequencies of 5–80 kHz were amplitude modulated by 20 Hz sine wave. A speaker for 5–40 kHz (SRS-3050A; Stax, Saitama, Japan) or 50–80 kHz (ES105A; Murata, Kyoto, Japan) was set 10 cm in front of the mice. Sound intensity was calibrated using the microphone (types 4135 and 2669; Brüel & Kjær, Nærum, Denmark) and the sound level meter (type 2610; Brüel & Kjær). The sound intensity was 60 dB sound pressure level (SPL) for flavoprotein fluorescence imaging and 80 dB SPL for two-photon imaging. The sound duration was 500 ms with a rise/fall time of 10 ms. The desired sound spectrum was determined using a digital spectrum analyzer (R9211A; Advantest, Tokyo, Japan) or the custom-written LabVIEW program. When the UF and DP (Stiebler et al. 1997) were activated specifically, frequency modulation (FM) direction-reversal stimulation (24 kHz/s) was used (Honma et al. 2013). The sound intensity was set to 60 dB SPL, and the band frequency was between 5 and 11 kHz.

#### Acoustic exposure.

**Fig. 9.**
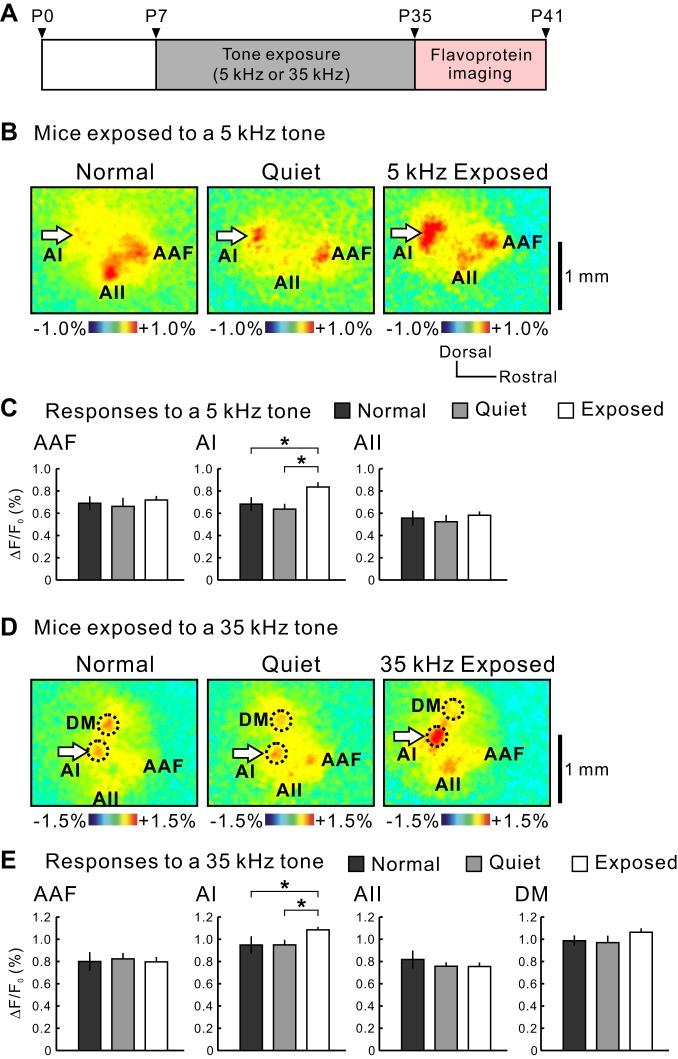
Plasticity after acoustic exposure in the AI and DM. *A*: schedule of experiments. Imaging was performed within 1 wk after cessation of acoustic exposure. P, period. *B* and *C*: response amplitudes to a 5-kHz tone in mice exposed to a 5-kHz tone (*P < 0.05, Mann-Whitney U-test; normal, *n* = 10; quiet, *n* = 6; exposed, *n* = 14). Mice rearing in a normal home cage (Normal), in the chamber without presentation of any tones (Quiet), or in the chamber with acoustic exposure (Exposed). *D* and *E*: response amplitudes to a 35-kHz tone in mice exposed to a 35-kHz tone (*P < 0.05, Mann-Whitney U-test; normal, *n* = 10; quiet, *n* = 6; exposed, *n* = 14).

**Fig. 10.**
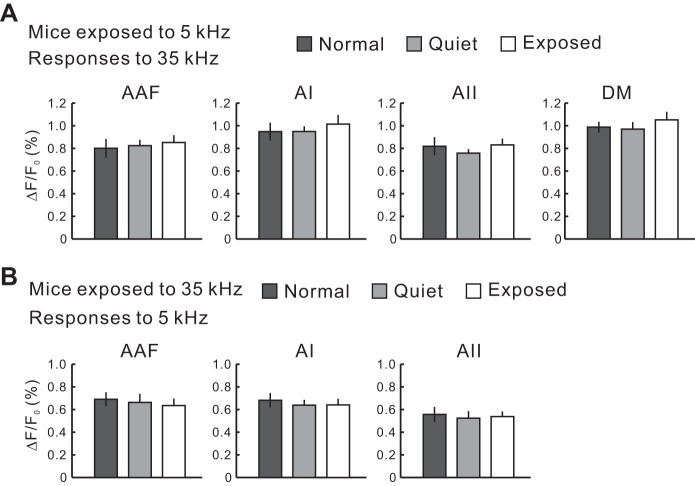
Frequency specificity in plasticity after acoustic exposure. *A*: the response amplitudes to a 35-kHz tone in mice exposed to a 5-kHz tone. There was no significant difference between normal and exposed or quiet and exposed mice. Normal, *n* = 10; quiet, *n* = 6; exposed, *n* = 14. *B*: the response amplitudes to a 5-kHz tone in mice exposed to 35-kHz. There was no significant difference between normal and exposed or quiet and exposed mice. Normal, *n* = 10; quiet, *n* = 6; exposed, *n* = 14.

For acoustic exposure experiments (see Figs. 9 and 10), home cages were placed in the sound-shielded chamber and exposed to a 5- or 35-kHz sound stimulus through a speaker (SRS-3050A; Stax) placed above the cage. The exposure sound consisted of an amplitude-modulated tone with a carrier frequency of 5 or 35 kHz and modulation frequency of 20 Hz. Duration of the tones was 500 ms, and a rise/fall time was 10 ms. The sound intensity was adjusted to 70 dB SPL at the floor of the cage. This tonal stimulus was repeated at 1 Hz throughout the exposing periods (P7–P35). Mice of normal groups were reared in the normal cages. Mice of quiet groups were reared in the chamber but not exposed to any tones. Flavoprotein fluorescence imaging was performed within 1 wk after exposure was finished. When a circular window was put on the response of the dorsomedial field (DM) to evaluate response amplitudes, a window was put to make the response amplitude maximum, kept >22 pixels apart dorsal to the AI response peak, according to the data (see Fig. 2*E*).

#### Retrograde tracer experiments.

To visualize neurons in the MGB projecting to each cortical region, a neural tracer was injected into the center of each region identified by flavoprotein fluorescence imaging (Horie et al. 2013). A glass capillary (tip diameter 20–30 μm) filled with tracer solution and a platinum wire was introduced into the center of the subregion of the right auditory cortex to ∼500 μm below the surface. Alexa Fluor 488- or 555-conjugated cholera toxin subunit B (CTB; Molecular Probes, Eugene, OR) was used in injections (see Fig. 13). Fluorescein and Texas Red (Molecular Probes) were used in some animals, but the results were the same. Fluorescent CTB solution (0.5% in phosphate buffer) was injected iontophoretically by a 5-μA pulse current (5 s on; 5 s off) for 15 min. In some experiments (see Figs. 8 and 12), biotinylated dextran amine (BDA; molecular weight 3,000; Molecular Probes) was injected iontophoretically by a 5-μA pulse current (7 s on; 7 s off) for 15 min. Survival of 3 days for fluorescent CTB or 7 days for BDA was ensured until perfusion. After anesthetizing mice deeply with pentobarbital (1.0 g/kg ip), the brains were dissected and immersed in 4% paraformaldehyde overnight, and a consecutive series of 40-μm-thick coronal or horizontal sections was cut using a sliding cryotome. To observe fluorescent tracers, sections were mounted on glass slides and covered with Fluoromount (Cosmo Bio, Tokyo, Japan).

To visualize BDA, sections were rinsed initially in 20 mM PBS and incubated in PBS containing 3% hydrogen peroxide and 0.1% Triton X-100 for 15 min at room temperature. After rinsing in 20 mM PBS containing 0.1% Triton X-100 (PBST), the sections were incubated for 40 min in 20 mM PBST containing avidin-biotin peroxidase complex (Vectastain ABC kit; Vector Laboratories, Burlingame, CA). Sections were rinsed in 20 mM PBS, and BDA was visualized in a solution comprising 0.05% diaminobenzidine tetrahydrochloride and 0.003% hydrogen peroxide in 50 mM Tris-HCl buffer (pH 7.4) for 20 min. All sections were finally, thoroughly rinsed in 50 mM Tris-HCl buffer and mounted onto gelatin-coated slides. Adjacent sections were counterstained using 0.1% cresyl violet (Chroma Gesellschaft, Kongen, Germany). After the mounted sections had dried, they were dehydrated in a graded ethanol series, cleared in xylene, and coverslipped using the covering reagent Bioleit (Okenshoji, Tokyo, Japan).

The borders of the MGB subdivisions were delineated, according to the SMI-32 immunolabeling pattern (Honma et al. 2013; Horie et al. 2013; LeDoux et al. 1985, 1987) and an atlas (Paxinos 2003; Paxinos and Franklin 2001; Paxinos et al. 2009). The immunohistochemistry of SMI-32 reacts with a nonphosphorylated epitope in neurofilament M and H (NNF). Sections were rinsed and incubated in PBST containing 3% hydrogen peroxide, as described for BDA visualization. After rinsing in 20 mM PBS, the sections were incubated overnight at room temperature with the monoclonal antibody (MAb) SMI-32 (1:2,000; Covance Research Products, Berkeley, CA) (Sternberger and Sternberger 1983), diluted with 20 mM PBS containing 0.5% skim milk. Sections were then incubated in anti-mouse IgG (1:100; MBL, Nagoya, Japan) at room temperature for 2 h. The sections were rinsed in 20 mM PBS, and the immunoreactions were visualized in a Tris-HCl buffer containing 0.05% diaminobenzidine tetrahydrochloride and 0.003% hydrogen peroxide for 5 min at room temperature. After visualization, the sections were coverslipped. All sections were observed under the light microscope (Eclipse Ni; Nikon, Tokyo, Japan) and a CCD camera (DS-Fi2; Nikon). The drawings and images were prepared using CorelDRAW (E Frontier, Tokyo, Japan), Illustrator (Adobe Systems, San Jose, CA), and Photoshop (Adobe Systems) software.

#### AAV injection to investigate interhemispheric connectivity.

To confirm that the corresponding subareas have interhemispheric connections with each other via the corpus callosum, adeno-associated virus (AAV) vector for GCaMP3 expression (Penn Vector Core, Philadelphia, PA) was injected into an area of the right AI after functional identification, which stains the axonal branches reaching the contralateral auditory cortex (see Fig. 3). A glass capillary (tip diameter: 20–30 μm) filled with AAV solution (500 μL) was introduced to a depth of 500 μm from the surface and slowly pressure injected for 30 min by a custom-made pump. After injection, the hole in the skull was covered with 2% agarose (1-B; Sigma-Aldrich), and the skin was sutured. Mice were recovered from anesthesia in their home cages. Two weeks after the injection, the left auditory cortex was observed using an epifluorescence microscope [excitation (Ex), 470–490 nm; emission (Em), 500–550 nm] and a two-photon microscope (Ex, 900 nm; Em, 500–550 nm).

#### Statistics.

The Mann-Whitney U-test or Wilcoxon signed-rank test was used to evaluate differences between unpaired or paired data from two groups, respectively. When data from three groups were compared, Tukey-Kramer test was used as a post hoc test after ANOVA. The correlation coefficients and the *P* values were calculated using Spearman rank correlation test. When the difference between two slopes was evaluated, the slope test was performed. The χ^2^ test was used to evaluate the difference between two percentages. These tests were performed using SPSS (IBM, Tokyo, Japan) or MATLAB software. All of the data in the text and graphs are presented as means ± SE.

## RESULTS

### 

#### Finding the new, distinct region identified from the AI.

Prior imaging studies have identified distinct auditory cortical regions based on response magnitudes to tones and the topographic organization of tone-frequency responses (cochleotopy) (Harel et al. 2000; Horie et al. 2013; Kubota et al. 2008; Sawatari et al. 2011; Takahashi et al. 2006). In this study, we try to confirm frequency organizations by demonstrating distinct response magnitude peaks and mirror-reversed frequency organization in the AAF and AI using flavoprotein fluorescence imaging. In addition, we try to observe flavoprotein fluorescence responses precisely within a cortical region, typically defined as the AI in mice, according to the Paxinos anatomic atlas (Paxinos and Franklin 2001). First, we observed neural responses in the right auditory cortex of C57BL/6 mice in a 5- to 70-kHz range after craniotomy (Fig. 2*A*). The frequency-organized map of the AI was arranged in a caudalrostral direction. Although only one area was activated in response to a 5-kHz tone, the AI responses to a 40-kHz tone were observed in two different areas, which were dorsoventrally separated from each other, as reported previously (Fig. 2*B*) (Issa et al. 2014; Tsukano et al. 2013a). We also confirmed that the AI high-frequency area showed two different response peaks when stimulated by high-frequency tones using transcranial flavoprotein fluorescence imaging (Fig. 2*C*). Significantly separated double peaks in ΔF/F_0_ were found by positioning serial ROIs across both dorsal and ventral AI high-frequency areas (Fig. 2*E*; *P* < 0.01 between ROI 6 vs. 11; *P* < 0.05 between ROI 11 vs. 20). These data confirmed the presence of apparently separated AI high-frequency areas in mice. The regions overlapped to the places where Stiebler et al. (1997) drew the UF and DP in their auditory cortical map, hereby referred to as Stiebler's UF and DP in this report, were confirmed by several studies (Honma et al. 2013; Joachimsthaler et al. 2014; Stiebler et al. 1997), and both areas are known to be activated by directional changes of slow FM sounds (Honma et al. 2013; Tsukano et al. 2013b). Moreover, cortical responses to ultrasonic sounds are not specific enough to divide the broad area into specific subareas. Therefore, we verified whether the dorsal high-frequency area of the AI overlaps Stiebler's UF or DP using FM directional changes. The dorsal area of the AI mapped between Stiebler's UF and DP (Fig. 2*D*). To evaluate these data quantitatively, we positioned ROIs across Stiebler's UF and DP, and the same ROIs were placed onto the image of the responses to a 35-kHz tone obtained from the same mouse (Fig. 2*F*). The response peaks of the dorsal area of the AI did not overlap Stiebler's UF or DP (Fig. 2*F*; *P* < 0.05 at ROI 9; *P* < 0.05 at ROI 21; *P* < 0.05 at ROI 31). The observation that the AI was divided into two areas in the response to high-frequency tones was also made in the left auditory cortex (data not shown). We visualized the auditory cortex in CBA/CaJ and Balb/c mice as well. The structures of responses of the AAF, AI, and AII were the same among three strains, and the AI was divided into two areas in response to high-frequency tones (data not shown). These results, along with prior studies, suggest that intrinsic response peaks identify functionally distinct regions in the auditory cortex (Kalatsky et al. 2005).

It remains unclear how auditory cortical regions, defined according to sound-response magnitudes, align with traditional cytoarchitectonic partitions. To address this problem, we investigated the cytoarchitectural pattern of the AI low-frequency area, the ventral AI high-frequency area, and the dorsal AI high-frequency area (see Fig. 4*A*) after identifying the regions by flavoprotein optical imaging. We characterized these areas histologically by immunolabeling NNF using MAb SMI-32, which is widely used to partition and identify various cortical and brain regions, including the auditory cortex (Budinger et al. 2000; Mellott et al. 2010; Ouda et al. 2012; Rothschild et al. 2010), MGB (Honma et al. 2013; Horie et al. 2013; Paxinos et al. 2009), visual cortex (Boire et al. 2005; van der Gucht et al 2001; Wong and Kaas 2009), and other regions. To perform this analysis, it is necessary to determine the low- and high-frequency areas of the AI on slice sections. However, mouse monoclonal SMI-32 immunolabeling using mouse IgG as a secondary antibody gave high-background staining after marking the identified area with injection of fluorescent beads, dye, or ink. Therefore, we injected BDA into an identified area of the AI in the right hemisphere in vivo and then observed NNF cytoarchitecture in the left AI on slice sections guided by axonal branches stained with BDA projecting from neurons in the right AI. It is widely known that the corresponding areas have interhemispheric projections via the corpus callosum in mammalian brains (Budinger et al. 2000; Oviedo et al. 2010; Rouiller et al. 1991; Xiong et al. 2012), as shown in the schema of Fig. 3*A*. We confirmed this fact in the present study as well (Fig. 3). After we identified the precise location of the subregions in the right auditory cortex using flavoprotein fluorescence imaging, we injected AAV-GCaMP3 solution into the AI 5-kHz area (Fig. 3, *B–D*). This technique visualizes the axon terminals originating from the injected subarea in vivo (Glickfeld et al. 2013; Oh et al. 2014). Two weeks after injection of AAV-GCaMP3 into the AI 5-kHz area in the right hemisphere, the fluorescent axon terminals were observed in the contralateral left AI 5-kHz area (Fig. 3*E*). GCaMP3-positive axon terminals were visualized using a two-photon microscopy in the place where an intense signal was observed in Fig. 3*E* (Fig. 3*F*), whereas no GCaMP3-positive terminals were observed outside of the AI (Fig. 3*G*). The large calcium responses to a 5-kHz tone derived from GCaMP3-positive terminals were observed in the left AI 5-kHz area (Fig. 3*H*), and the responses of the AAF and AII were close to those observed in a naïve mouse (Fig. 3*D*). These data confirm that it is possible to observe the NNF pattern of the identified area in the contralateral auditory cortex on slice sections, guided by axon terminals, stained by BDA injected into the right auditory cortex.

The NNF immunolabeling pattern appeared much denser in the dorsal part than in the ventral part of the AI high-frequency area or the AI low-frequency area, whereas the weak NNF immunolabeling pattern appeared in the latter two areas (Fig. 4*B*). Moreover, the dorsal part of the AI high-frequency area appeared to have the particular laminar pattern of immunolabeling. To analyze quantitatively the difference in NNF immunolabeling patterns, we first counted the number of NNF-positive neurons in each area. However, labeled neurons were not significantly different in number among the three areas (Fig. 4*C*). NNF immunolabeling clearly distinguished pyramidal from nonpyramidal neurons, the two most common neuron types. Pyramidal neurons had NNF-positive apical dendrites, reaching the superficial layer, and a densely labeled large soma (Fig. 4*D*). Conversely, nonpyramidal neurons had few NNF-positive apical and basal dendrites, and their soma was labeled weakly. Therefore, the difference in the ratios of NNF-positive pyramidal neurons to nonpyramidal neurons could account for the apparent density difference with the NNF immunolabeling patterns. In *layers II*, *III*, and *IV*, the ratio of NNF-positive pyramidal/nonpyramidal neurons did not differ noticeably, and nonpyramidal neurons were dominant in all three areas (Fig. 4*E*). However, the deeper *layers V* and *VI* in the dorsal AI high-frequency area were almost fully occupied by NNF-positive pyramidal neurons. The ratio of NNF-positive pyramidal/nonpyramidal neurons was significantly higher than that observed in the other two areas [Fig. 4*F*; *P* < 0.05 (*area a* vs. *c*); *P* < 0.05 (*area b* vs. *c*)] when compared in *layers V*, *VI*, and *II–VI*, whereas there was no significant difference between *areas a* and *b* in any layer. Thus the presence of numerous NNF-positive pyramidal neurons in the deeper layers could explain the dense NNF immunolabeling pattern in the dorsal AI high-frequency area, since these neurons have thick NNF-positive basal dendrites and apical dendrites that reach superficial layers. These data (Figs. 2 and 4) strongly suggest that the dorsal AI high-frequency area, located between Stiebler's UF and DP, is likely a newly defined region with a different cytoarchitectural pattern. We tentatively refer to this new area as the DM (Fig. 1*D*).

#### Frequency organizations of the AI and DM.

We next re-evaluated the precise frequency organizations of the auditory cortex based on the finding that the dorsal AI high-frequency area is a newly defined region. We investigated frequency gradients by plotting the response peaks using flavoprotein fluorescence imaging described in our previous study (Fig. 5) (Honma et al. 2013). The posterior frequency-organized region labeled as the “AI,” has a low-to-high (5–80 kHz) tone-frequency response axis directed from the dorsocaudal to the ventrorostral temporal cortex (Fig. 5, *A* and *B*). The frequency gradient of the AI ran in a mild clockwise direction. The corresponding frequency gradient for the newly identified DM was directed from ventrocaudal to dorsorostral anatomic axis (Fig. 5, *A* and *C*). Similar maps were obtained using other sound-intensity levels (for example, at 40 dB SPL; data not shown). The slopes of the AI and DM frequency gradients were not significantly different (*P* > 0.9) using comparison of regression coefficients. Both the AI and DM have high-frequency bands inside of their frequency gradients. The ratios of the response amplitudes of the DM and AI were plotted against the stimulus frequency to investigate whether the response property was similar in these two regions (Fig. 5, *D* and *E*). Although the AI is responsive to ultrasonic sounds, the DM is more responsive to ultrasonic sounds over 40 kHz than is the AI (Fig. 5*E*). These data suggest that the DM and AI have distinct frequency gradient directions for tone-response topographies and that the DM responds more vigorously to high-frequency sounds than the AI.

Differences in robustness of frequency gradients between regions have been confirmed using two-photon imaging (Issa et al. 2014). We have shown that the DM and AI have a robust, frequency-organized structure using flavoprotein fluorescence imaging. To evaluate the extent of heterogeneity in the frequency organization at the high-resolution scale, we performed in vivo two-photon imaging to determine the properties of neurons in the supragranular layers in the AI and DM (Fig. 6), since the regional characteristic obtained by low-resolution imaging reflects an ensemble of the single neuronal properties in *layers II/III* (Andermann et al. 2011; Marshel et al. 2011; Tohmi et al. 2014). After identifying the precise location of the AI or DM using flavoprotein fluorescence imaging (Fig. 6*A*), we injected Fura-2 solution to observe the calcium responses in each neuron (Fig. 6*B*). The frequency at which the response amplitude reached the maximum in a given neuron was defined as the BF (Fig. 6*C*). Although the overall frequency gradients in the DM and AI had the same directions as those defined using flavoprotein fluorescence imaging, the frequency gradient in the DM neurons was less variable than that in the AI (Fig. 6, *D* and *E*). The degree of the frequency organization of the microscopic structures was evaluated using residuals between each neuron and the regression line. The value of residuals was greater in the AI than in the DM (Fig. 6*F*; *P* < 0.001), implying that neuronal responses in *layers II/III* of the AI were more complex than those in the DM.

The DM was less sensitive to low-frequency tones than the AI in flavoprotein fluorescence imaging (Fig. 5*E*). This was explained by the neuronal distribution revealed by two-photon imaging; there were few neurons in the DM with BF <20 kHz (Fig. 6, *G* and *H*). In contrast, the bandwidth of the neurons in the DM and AI was similar (Fig. 6*I*). A tendency that bandwidth values obtained in two-photon studies, including this study, are smaller than those in electrophysiological study (Guo et al. 2012) might be attributed to the property of a calcium-sensitive dye in detecting spikes.

#### Frequency organizations of the AAF.

We also found the presence of the precise frequency organization in the AAF (Fig. 7). The neural response to a 5-kHz tone was placed at the most rostral part of the AAF. When mice heard tones at 5–80 kHz, neural responses to higher tones shifted to the ventrocaudal direction. No clear response was found near Stiebler's UF indicated by the white arrow (Fig. 7*A, inset*). The plotting of response peaks elicited by tones at 5–80 kHz also confirmed the presence of the precise octave-based frequency organization in the AAF (Fig. 7, *B* and *C*), as reported previously (Horie et al. 2013; Issa et al. 2014). Therefore, the low-high and high-low mirror-imaged frequency organizations between the AAF and AI, reported in other species, were preserved in mice as well.

There is a discrepancy in the direction of the frequency organization of the AAF between studies using optical imaging (Fig. 7) (Honma et al. 2013; Issa et al. 2014) and studies using electrophysiology (Guo et al. 2012). The latest mapping study using electrophysiology considered the whole area, which was composed of the AAF, Stiebler's UF, and the DM in this study as a single region of the AAF, as shown by the schema in Fig. 8*A* (Guo et al. 2012), and the direction of the frequency organization of the AAF was drawn dorsocaudally (Fig. 1*C*). Therefore, we investigated NNF immunolabeling patterns and anatomical properties in the AAF, Stiebler's UF, and the DM (Fig. 8, *B*–*E*) to confirm our physiological mapping shown in Fig. 7. First, NNF immunolabeling patterns of the AAF and Stiebler's UF were totally different (Fig. 8*C*); apparently, the gross immunolabeling pattern of Stiebler's UF was much weaker than that of the AAF (Fig. 8*C*). Although there was no difference in number of total NNF-positive neurons between the AAF and Stiebler's UF (Fig. 8*D*), Stiebler's UF lacked NNF-positive neurons in the *layer VI* (Fig. 8*E*), and the density of NNF immunolabeling within somas and dendrites per se was much weaker in Stiebler's UF, especially in the *layer III*, shown in Fig. 8*C*. Furthermore, the density of NNF immunolabeling in the DM was denser than that in the AAF (Figs. 4*B* and 8*C*), which is because the DM contained more than twice as many NNF-positive neurons as did the AAF and Stiebler's UF (Fig. 8*D*). These data indicate that the area that was defined as the AAF in the past study (Guo et al. 2012) (Fig. 8*A*) includes three subregions with a distinct immunolabeling pattern.

The Stiebler's UF is more responsive to slow FM stimuli than to tones and does not have any frequency-organized structure (Honma et al. 2013; Stiebler et al. 1997), suggesting that the Stiebler's UF belongs to the belt region that receives thalamic inputs from the dorsal division of the MGB (MGd) (Winer and Schreiner 2011). Therefore, we investigated from which division of the MGB the Stiebler's UF receives dense projections (Fig. 8*F*). We injected BDA solution into the Stiebler's UF as a retrograde tracer after identifying the region, according to the response to FM reversal stimuli (Fig. 2*D*), and evaluated neuronal distribution using coronal sections between 2.8 and 3.4 mm posterior to the bregma. The MGv and MGd were partitioned according to the SMI-32 immunolabeling patterns (Honma et al. 2013; Horie et al. 2013; LeDoux et al. 1985, 1987) and an atlas (Paxinos 2003; Paxinos and Franklin 2001; Paxinos et al. 2009). Whereas 64.6% neurons of BDA-stained neurons were placed in the MGv, as much as 35.4% neurons were placed in the MGd (Fig. 8*F*). These data are clearly consistent with the results reported by Hofstetter and Ehret (1992), showing that the Stiebler's UF received robust thalamic projections from both MGd and MGv. In contrast, as for the region referred to as the AAF in the present study, 90% of the thalamic inputs originated from the MGv, and only 10% originated from the MGd (Fig. 8*F*), which is consistent with the fact that the AAF is one of the lemniscal core regions. Furthermore, we injected BDA solution into the DM to clarify the origin of the thalamic input. The DM presumably receives thalamic information from the MGv but not from the MGd, because the DM has the clear frequency organization shown in Fig. 5. As expected, it was revealed that as much as 97% neurons projecting to the DM were localized in the MGv, and remaining 3% neurons were located in the MGd (Fig. 8*F*). Overall, these data indicate that the AAF, Stiebler's UF, and DM are not combined into a single region but three different regions with distinct histological and anatomical properties. Hence, these data support our physiological mapping that reveals that the frequency gradient of the AAF travels ventrocaudally (Fig. 7).

#### Comparison of the effects of acoustic exposures between the AI and DM.

The number of cortical neurons with a particular characteristic frequency increases after animals are passively exposed for a long time to the tonal stimuli at the same frequency (de Villers-Sidani et al. 2007; Nakahara et al. 2004; Zhang et al. 2001). This plastic change can be observed, as response amplitude increases when observed by flavoprotein fluorescence imaging (Takahashi et al. 2006; Tohmi et al. 2009). The potentiation induced by the passive acoustic exposure exhibits regional and tonal specificity. Whereas the AAF is relatively insensitive to the passive acoustic exposure (Takahashi et al. 2006), the AI responses are clearly potentiated by the exposure (de Villers-Sidani et al. 2007; Nakahara et al. 2004; Zhang et al. 2001). We tested whether the plastic change to acoustic exposure was observed in the DM, which is expected to have different properties from those in the AI. We observed that when mice were reared under 5 kHz tone exposure from P7 to P35 (Fig. 9*A*), significant potentiation of neural responses to a 5-kHz tone occurred only in the AI (ΔF/F_0_ in normal environment, 0.68 ± 0.06%, *n* = 10; quiet environment, 0.64 ± 0.05%, *n* = 6; exposed, 0.87 ± 0.04%, *n* = 14), and such potentiation was not found in the AAF or AII (Fig. 9, *B* and *C*). In the AAF, AI, AII, and DM, the responses to a 35-kHz tone were not clearly potentiated after exposure to a 5-kHz tone because of the frequency specificity in the potentiation (Fig. 10*A*), as reported previously (Takahashi et al. 2006). When mice were exposed to a 35-kHz tone, significant potentiation was observed only in the AI (normal environment, 0.95 ± 0.08%, *n* = 10; quiet environment, 0.95 ± 0.05%, *n* = 6; exposed, 1.09 ± 0.03%, *n* = 14), whereas no potentiation was observed in the DM as in the AAF or AII (Fig. 9, *D* and *E*). Although the size of responsive areas after the plasticity occurred seemed to be expanded in an experience-dependent manner, the location of the AI peak was not shifted by the plasticity. Therefore, it is unlikely that the AI and DM have merged by the plasticity. The neural responses were not potentiated in all three areas to a 5-kHz tone (Fig. 10*B*). Flavoprotein fluorescence imaging showed that responses of the DM to a 5-kHz tone were very weak and diffused and did not show robust, clear peaks in response to a 5-kHz tone (Fig. 2*B*). Moreover, single neuronal analysis showed that there were few low-frequency neurons in the DM (Fig. 6). Therefore, reliable analysis was not possible regarding DM responses to a 5-kHz tone, and we used only a 35-kHz tone to evaluate the effect of acoustic exposures. Overall, the resistance to plasticity of the DM indicates that the DM is a functionally distinct region from the AI.

#### Thalamocortical projections from the MGB to the DM.

**Fig. 11.**
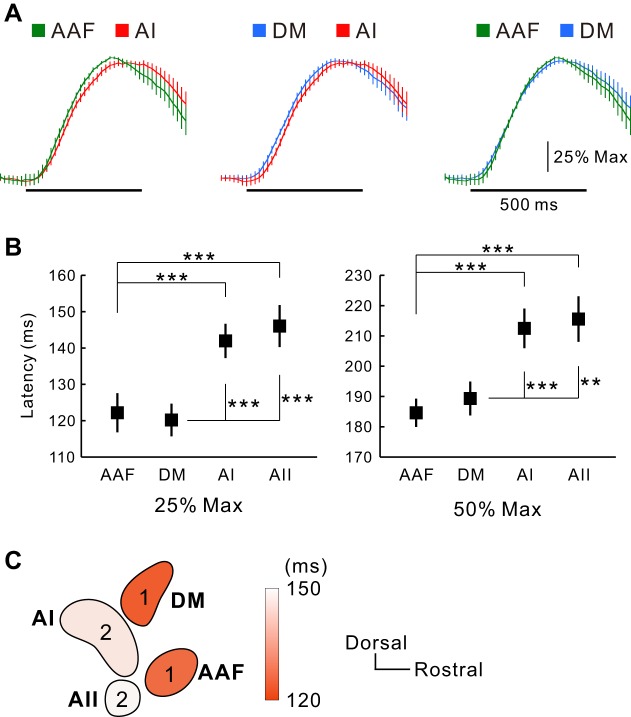
Responses in the DM and AAF faster than those in the AI and AII. *A*: temporal profiles of ΔF/F_0_ in the AAF, AI, and DM in response to a 35-kHz tone. Frame rate was set at 54 Hz. Horizontal black bars indicate the stimulus period. *B*: times required to reach 25% (*left*) and 50% (*right*) of the maximal ΔF/F_0_ after the stimulus onset in the AAF, DM, AI, and AII (***P < 0.001, **P < 0.01, 1-way repeated-measures ANOVA; *n* = 18 each). *C*: schema of latencies reaching 25% maximum by color. The numbers indicate the order of response.

Auditory cortical regions have different latencies to auditory stimuli across species. In previous studies, neurons in the AAF were activated with shorter latencies than those in the AI (Kubota et al. 2008; Linden et al. 2003; Sawatari et al. 2011). We verified the latency of the neural response to a 35-kHz tone in the DM using flavoprotein fluorescence imaging (Fig. 11). The latency was evaluated as the time until the fluorescence signal reached 25% or 50% maximum of the peak. The latency of the AAF response to a 35-kHz tone was shorter than that of the AI, as we described previously (Fig. 11*A*) (Kubota et al. 2008), which is consistent with the results obtained by voltage-sensitive dye imaging (Sawatari et al. 2011). The latency of the DM was also shorter than that of the AI and comparable with that in the AAF (Fig. 11*A*). The latencies to reach 25% maximum were 122.2 ± 5.4 ms in the AAF, 120.2 ± 4.5 ms in the DM, 142.0 ± 4.7 ms in the AI, and 146.0 ± 5.8 ms in the AII (*n* = 18, each; Fig. 11*B*). The latencies to reach 50% maximum were 185 ± 4.7 ms in the AAF, 189.3 ± 5.6 ms in the DM, 212.5 ± 6.5 ms in the AI, and 215.6 ± 7.5 ms in the AII (*n* = 18, each; Fig. 11*B*). The latencies to reach 25% and 50% maximum had the same tendency. The latencies of the AAF and DM were significantly shorter than those of the AI and AII, and there were no significant differences between the AAF and DM or the AI and AII (Fig. 11*C*). These data also support the fact that the DM receives thalamocortical projections directly from the MGv but not via the AAF or AI.

**Fig. 12.**
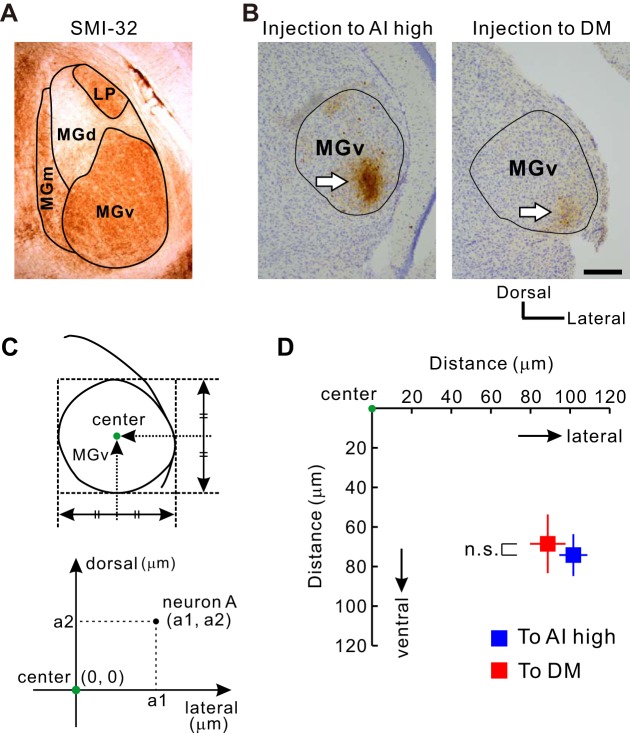
Distribution of MGv neurons projecting to the AI or DM in coronal sections. *A*: regional subdivisions of the MGB parcellated using SMI-32. LP, lateral posterior nucleus; MGm, medial division of MGB. *B*: neurons in the MGB projecting to the auditory cortex in coronal sections. BDA was injected iontophoretically into the AI high-frequency area or the DM. BDA-positive MGB neurons projecting to the AI high-frequency area are located in the ventrolateral part in the MGv (*left*). BDA-positive MGB neurons projecting to the DM are also located in the ventrolateral part in the MGv (*right*). White arrows indicate where BDA-positive neurons are assembled. Scale bar, 200 μm. *C* and *D*: quantitative analysis. The center of the MGB was defined as (0, 0). The relative location of each neuron was measured, and the coordinates were averaged (*D*). Neurons projecting to the DM were localized in the lower half of the MGv and at the same level as the region projecting to the AI high-frequency region dorsoventrally (n.s., *P* > 0.9, Mann-Whitney U-test; 131 neurons projecting to the AI in 7 mice; 156 neurons projecting to the DM in another 7 mice).

**Fig. 13.**
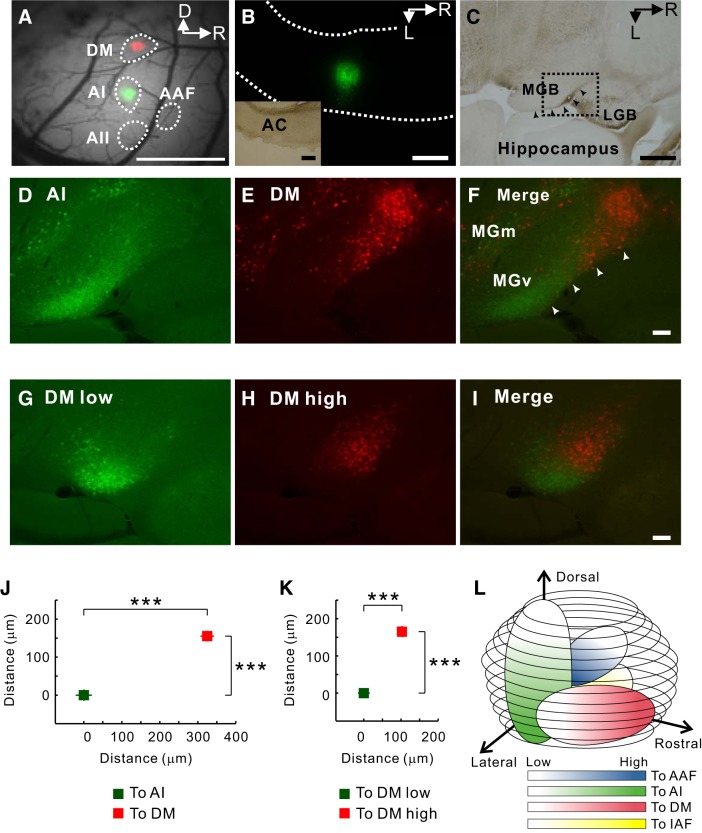
Distribution of MGv neurons projecting to the DM in horizontal sections. *A*: injection sites in the AI and DM. Image of the brain surface and merged images obtained through a green or a red filter. The dotted lines indicate the outlines of responses to a 40-kHz tone. Scale bar, 1 mm. D, dorsal; R, rostral. *B*: injection site after a horizontal slice was prepared. The dotted lines indicate the outlines of the auditory cortex. *Inset*: the same image observed under bright-field microscopy. Scale bars, 500 μm. L, lateral. *C*: bright-field view of the MGB in a horizontal slice. The dotted rectangle represents the window containing images of *D–F*. Scale bar, 500 μm. LGB, lateral geniculate body. *D–F*: neurons projecting to the AI ventral high-frequency area and DM (*F*). The arrowheads indicate the lateral edge of the MGv as shown in *C*. Scale bar, 100 μm. *G–I*: neurons projecting to the low- and high-frequency areas of the DM (*I*). Scale bar, 100 μm. *J*: the relative coordinates of neurons projecting to the DM vs. the AI. The average of the coordinates of neurons projecting to the AI was set as the origin. In 6 mice, 71 neurons projecting to the AI and 103 neurons projecting to the DM were found. ***P < 0.001, Mann-Whitney U-test. *K*: the relative coordinates of neurons projecting to a low-frequency (25 kHz) area vs. high-frequency (60 kHz) area in the DM. The average of the coordinates of neurons projecting to a low-frequency area was set as the origin. In 3 mice, 33 neurons projecting to the low-frequency area of the DM and 28 neurons projecting to the high-frequency area were found. ***P < 0.001, Mann-Whitney U-test. *L*: 3-dimensional schematic view of the relative locations of 4 compartments within MGv. The schema of the compartment projecting to the insular auditory field (IAF), AAF, or AI was compensated, according to the elegant study by Takemoto et al. (2014) and, in part, the study by Horie et al. (2013).

The mouse MGv is composed of several compartments that topographically project to their corresponding subregions in the auditory cortex (Horie et al. 2013; Takemoto et al. 2014). The region that projects to the AAF or AI is localized in the middle part of the MGv. On these bases, we tried to identify the region that projected to the DM. First, we injected BDA into the AI or DM after identification, and we evaluated the locations of neurons that project to the AI or DM using coronal sections (Fig. 12). Neurons projecting to the DM were found in the ventral half of the MGv, and those projecting to the ventral AI high-frequency area were observed in a neighboring region located at the same ventrodorsal level (Fig. 12). However, we found that neurons projecting to the DM were located rostral to those projecting to the AI as a whole. Therefore, we prepared horizontal sections to evaluate the relative rostrocaudal location of neurons projecting to the DM and AI (Fig. 13). We injected Alexa Fluor-conjugated CTB into the ventral AI high-frequency area or the DM, identified using a tone at 40 kHz (Fig. 13, *A* and *B*). In horizontal sections of the MGv, the neurons projecting to the DM were located more rostrally than the neurons projecting to the AI (Fig. 13, *C*–*F*). The relative location of the neuronal population projecting to the DM was measured in reference to the averaged coordinates of neurons projecting to the AI (Fig. 13*J*). The neurons projecting to the DM were located significantly more rostral and more medial compared with those projecting to the AI (*P* < 0.001, for both).

Next, we tested whether the frequency organization in the DM reflects the distinct frequency organization in a single region within the MGv or partly overlapped regions projecting to the AI and DM, as suggested by the fork-shaped, frequency-organized map proposed by Issa et al. (2014) (Fig. 1*C*). We injected fluorescent CTB into the low- and high-frequency regions of the DM. Results clearly indicated that the frequency organization in the MGv projecting to the DM was structured along a single latero-medial axis within a single compartment of the MGv (Fig. 13, *G–I*). The quantitative distribution map showed a significant place shift in location between the areas projecting to the low- and high-frequency areas of the DM (Fig. 13*K*; *P* < 0.001). These data indicate that neurons projecting to the DM are localized in the rostral part of the MGv with a distinct frequency organization along the latero-medial axis, and no fewer than four compartments were independent from each other within the MGv (Fig. 13*L*).

#### Vocalization processing in the auditory cortex.

**Fig. 14.**
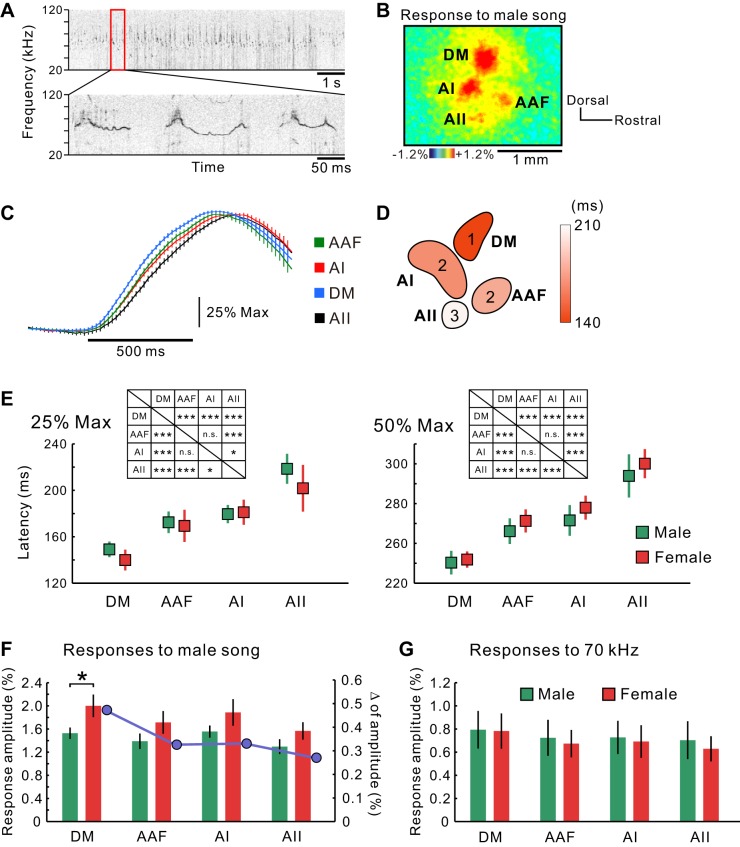
Auditory responses to courtship male songs. *A*: recorded spectrogram of a courtship song produced by a male C57BL/6 mouse when approaching a female (*top*). The red box indicates the segment used in this study as a stimulus (*bottom*). *B*: the typical image of the response in the auditory cortex to the male's song. The dorsal anterior “UF” region identified by Stiebler et al. (1997) was not activated, probably because this region is responsive to slow FM sweeps, ∼24 kHz/s, which were not included in the male's songs. The DM was identified based on the responses to a 70-kHz tone, as performed in the previous sections. The response peaks of the DM to a 70-kHz tone and a courtship song did not shift significantly. *C*: temporal profiles of ΔF/F_0_ in each region in response to the male's song (*n* = 20). Images were obtained at 54 Hz. *D*: schema showing the latency of responses to reach 25% maximum. *E*: the time required to reach 25% (*left*) or 50% maximum (*right*); male, *n* = 7; female, *n* = 13. *Insets*: statistical significance of pair-wise comparisons between regions with respect to latency required to reach 25% maximum (*left*) and 50% maximum (*right*) of the response peak to a male's song. The sexes were mixed, because there was no difference between them. ***P < 0.001, *P < 0.05, 1-way repeated-measures ANOVA; *n* = 20. *F*: response amplitudes to a male's song (*left* axis). The blue plots indicate the difference in averaged amplitudes between females and males (*right* axis); male, *n* = 15; female, *n* = 16. *P < 0.05, Mann-Whitney U-test. *G*: response amplitudes to a 70-kHz ultrasonic tone; male, *n* = 15; female, *n* = 16.

Although the frequency gradient in the AI also includes ultrasonic bands, the responses of the DM to ultrasonic tones were larger than those of the AI (Fig. 5*E*). Therefore, we verified the possibility that the DM may be involved in ultrasonic courtship songs produced by male mice for females (Fig. 14*A*). Although this courtship vocalization produced by male mice is one of the representative ultrasonic, communication-related sounds (Asaba et al. 2014; Hammerschmidt et al. 2009; Holy and Guo 2005), as well as isolation calls produced by pups (Ehret and Haack 1982; Thornton et al. 2005; Uematsu et al. 2007), no research has been performed about central processing of courtship songs, although several studies about pup isolation calls have been reported (Galindo-Leon et al. 2009). The courtship songs produced by male mice include characteristic features, such as a high-frequency band over 50 kHz, fast FM, pitch jumps, and intermittent rhythms (Fischer and Hammerschmidt 2011; Lahvis et al. 2011). The AAF, AI, AII, and DM in both sexes were clearly activated by stimulation with songs produced by males (Fig. 14*B*). Interestingly, the temporal order of the rising phase of neural responses to a male's courtship song was different from that of an artificial tone (Fig. 14, *C* and *D*). The DM was activated first, followed by simultaneous activation of the AAF and AI, and the AII was activated last. The latency to reach 25% maximum was 143 ± 6.2 ms in the DM, 170 ± 9.4 ms in the AAF, 181 ± 7.4 ms in the AI, and 208 ± 13.7 ms in the AII (Fig. 14*E*), and the latency to reach 50% maximum was 243 ± 6.5 ms in the DM, 279 ± 8.7 ms in the AAF, 291 ± 9.3 ms in the AI, and 340 ± 11.9 ms in the AII (*n* = 20 in total; male, *n* = 7; female, *n* = 13; Fig. 14*E*). As for the latency, there were no differences between male and female mice in all of the regions. These data indicate that vocalization is similarly processed in the DM of both male and female mice regarding the latency of the DM responses to a male's courtship song. In contrast, the amplitudes of the response to a male's song were significantly larger in females than in males in the DM alone (Fig. 14*F*; male, 1.61 ± 0.10%, *n* = 15; female, 2.0 ± 0.19%, *n* = 16; *P* < 0.05). The response amplitudes in the AAF, AI, and AII were slightly larger in females than in males, although there were no significant differences between the sexes (AAF, *P* > 0.6; AI, *P* > 0.8; AII, *P* > 0.5). The slight difference in the AAF, AI, and AII might be attributed to the significant difference in the DM, as it was reported that neural responses in a downstream region with a longer latency are affected by neural responses in an upstream region with a shorter latency in the auditory cortex (Kubota et al. 2008). As expected from this idea, there was no difference between the sexes in response to a 70-kHz artificial tone (Fig. 14*G*). These data imply that ultrasonic courtship songs produced by males, mediated via a distinct rostral compartment of the MGv, reach the DM first and are processed further through pathways from the DM to other cortical regions in mice.

## DISCUSSION

In the present study, we observed the regional characteristics in the mouse auditory cortex using flavoprotein fluorescence imaging, two-photon calcium imaging, immunohistochemistry, and tracer experiments. The regional borders of the AI were re-delineated by isolating the DM, which has been reported to be a part of the AI, specialized for processing high-frequency sound signals in mice (Guo et al. 2012; Issa et al. 2014; Joachimsthaler et al. 2014; Sawatari et al. 2011; Stiebler et al. 1997). In the present study, we identified the DM as the fifth frequency-organized region in the cortex, in addition to the three frequency-organized regions—the AAF, AI, and AII—in the auditory cortex (Issa et al. 2014; Kubota et al. 2008) and the insular auditory field (IAF) in the insular cortex (Gogolla et al. 2014; Sawatari et al. 2011; Takemoto et al. 2014). We demonstrated that the frequency gradient of the AI ran from the dorsocaudal to ventrorostral direction, whereas the frequency gradient in the DM ran from the ventrocaudal to dorsorostral direction. The direction of the frequency organization of the AAF was from dorsorostral to ventrocaudal, consistent with recent reports (Horie et al. 2013; Issa et al. 2014; Kubota et al. 2008). The DM responses to a courtship song by males were significantly larger in female mice, which is the first evidence to suggest that the gender-specific biological importance of courtship songs might be reflected in the properties of the mouse auditory system.

### 

#### Technical merits of using flavoprotein fluorescence imaging to investigate cortical functions in mice.

There were several technical advantages in the present study to localize the newly found DM region. First, we used flavoprotein autofluorescence imaging to visualize the mouse auditory cortex. Flavoprotein fluorescence imaging gave us very similar results to those obtained in GCaMP3-expressing mice (Issa et al. 2014) regarding the regional borders between the DM and surrounding regions. This method detects neural activity based on activity-dependent oxygen metabolism; therefore, there is no need to stain neurons using dye solutions, thus allowing uniform observability on the cortical surface (Llano et al. 2009; Shibuki et al. 2003, 2006) and allowing the delineation of regional borders, as in our previous studies (Honma et al. 2013; Kubota et al. 2008; Ohshima et al. 2010). Because of the merits of intrinsic fluorescence imaging, we were able to find a small trough in neuronal activity between the AI and DM (Fig. 2), as seen in GCaMP3-expressing mice (Issa et al. 2014). A critical requirement for delineating small cortical regions is the uniform distribution of fluorophores in the brain, which was based on endogenous flavoproteins or homogenously expressed GCaMP3. In contrast, unit recording requires the insertion of an electrode into the cortex, and therefore, the recording sites must be set at some intervals. Therefore, the regional border, such as that between the AAF and AI, must be defined as a result of the assumption that the border corresponds to the frequency-gradient reversal line based on unit recording described in previous studies. Thus it is no wonder that the small trough between the DM and the ventral AI high-frequency area was not found in previous studies using unit recording. The DM is sensitive to ultrasonic sounds over 40 kHz, and the response of the AI is somewhat weaker than that of the DM. These factors might have led to the conclusion that a single frequency gradient extended from the AI low-frequency area to the DM in previous electrophysiological experiments (Guo et al. 2012) or that the strong frequency gradient traveled across these two different regions (Issa et al. 2014).

Another benefit of flavoprotein fluorescence imaging is that variability of the signal amplitudes in ΔF/F_0_ is very small in each mouse, especially when transcranial imaging is performed. Because the skull of a mouse is transparent, and flavoproteins exist originally in neurons, we can observe the same pattern of responses transcranially without craniotomy, which might confound the results. Moreover, as flavoproteins are an endogenous protein in the mitochondrial electron transport chain, it is very unlikely that flavoproteins work as a calcium chelator and have some artificial effects on the calcium dynamics, which is essential for the induction of cortical plasticity (Zucker 1999).

Finally, we were able to combine flavoprotein fluorescence imaging with anatomical techniques using fluorescent traces, because the flavoprotein signals were much weaker than those from the fluorescent tracers. The combination of flavoprotein fluorescence imaging and microinjection of a retrograde fluorescent tracer could reveal multiple compartments in the MGv. Because the MGv is located deep within the brain, it is difficult to perform precise MGv experiments without anatomical studies. Direct optical imaging to observe responses in the MGv has not been reported. Although functional MRI (fMRI) imaging can be used to observe activities deep within the brain, the spatial resolution is too low to find multiple compartments in the mouse MGv. Without the fine spatial resolution achieved by anatomical studies, the precise multicompartments with a distinct frequency gradient (Fig. 13*L*) could not be visualized, because the general concept of one frequency organization in one region is also applicable in mice. The combination of optical imaging and localized tracer injection into functionally identified, small cortical regions is expected to be greatly advantageous for detecting fine topological organization in other sensory cortices other than the auditory cortex.

#### New definition of regions included in the mouse auditory cortex.

Our findings suggest that the term UF might not be appropriate. Stiebler et al. (1997) defined the UF as a distinct, frequency-unorganized region, where neurons have a characteristic frequency over 50 kHz (Fig. 1*A*). Then, UF as a distinct region was denied, and the term UF has also been used to indicate the high-frequency area of the AAF and AI (Fig. 1*B*) (Guo et al. 2012) or only the dorsal AI high-frequency area (Fig. 1*C*) (Issa et al. 2014). However, optical imaging revealed that frequency direction of the AAF runs ventrocaudally (Issa et al. 2014) and that the AII has the distinct frequency organization (Issa et al. 2014; Kubota et al. 2008). By delineating the DM, we have identified the four distinct frequency-organized regions of the AAF, AI, DM, and AII, in total, each of which has its own high-frequency area (up to 80 kHz) corresponding to the UF. Optical imaging also revealed that a vacant region insensitive to pure tones containing vocalization-specific neurons is located between the AAF and AI dorsal high-frequency area (Issa et al. 2014), and we have already reported that the region between the AAF and DM is responsive to slow FM components, regardless of tonal frequency range (Fig. 1*D*) (Honma et al. 2013; Tsukano et al. 2013b). These facts indicate that the distinct FM-sensitive region exists in this area in the mouse auditory cortex, similar to that observed in bats (Suga and Jen 1976). Actually, there is a possibility that Stiebler et al. (1997) might perform recordings without distinguishing between the DM and frequency-unorganized, FM-sensitive region. They mentioned in their paper that neurons observed in the rostral part of the UF were FM sensitive but not tone sensitive (Stiebler et al. 1997). The confusion of two different regions might obscure a frequency-organized structure and lead to the misconception that UF was not frequency organized (Stiebler et al. 1997). Overall, we propose to subdivide the dorsal part of the auditory cortex precisely and to assign new, anatomical names: the dorsoanterior field (DA) to the FM-sensitive region just dorsal to the AAF and the DM to the region newly identified in the present study (Fig. 1*D*), in addition to the classical DP named previously (Stiebler et al. 1997).

Multiple frequency organizations have been found in various mammals. By identifying the DM with a distinct frequency gradient using flavoprotein fluorescence imaging, multiple frequency organizations were revealed in the mouse auditory cortex in the present study. In primates, frequency-organized structures exist in the belt region besides the core region. With the use of fMRI, it was revealed that the caudal-medial (CM) field in macaques, which belongs to the belt, has stronger frequency gradient than do fields in the core, and other belt fields also have a frequency gradient (Petkov et al. 2006). Moreover, multiple frequency-organized structures were also found in rodents, such as guinea pigs (Nishimura et al. 2007), ferrets (Bizley and King 2009), chinchillas (Harel et al. 2000), and rats (Higgins et al. 2010; Kalatsky et al. 2005) using optical imaging. Especially the auditory cortical maps revealed in rats (Higgins et al. 2010; Kalatsky et al. 2005) are quite similar to those we revealed in mice. Several imaging studies have revealed that the AII is frequency organized in mice (Issa et al. 2014; Kubota et al. 2008). The IAF in the mouse insular cortex anterior to the auditory cortex is also frequency organized (Sawatari et al. 2011; Takemoto et al. 2014). Hence, at least five frequency-organized regions exist inside and near the auditory cortex in mice. Each frequency-organized region receives distinct thalamic information from equivalent compartments with a distinct frequency organization in the MGv (Fig. 13*L*) (Horie et al. 2013; Takemoto et al. 2014), and the sound responses are presumably conveyed through the course of the hierarchical, inter-regional processing in the auditory cortex (Fig. 14) (Kaas and Hackett 2000; Kubota et al. 2005).

The basic concept of “core and belt” prevails in the auditory cortex across species (Winer and Schreiner 2011). The core region contains the AAF and AI, which receive dense projections from the frequency-organized MGv and process lemniscal information from the cochlea. The belt region surrounding the core receives nonlemniscal information from the frequency-unorganized MGd across species basically (Winer and Schreiner 2011). Although the DM in mice is located dorsal to the AAF and AI and should be considered as the belt (Fig. 1*D*), the DM has a clear frequency gradient and receives topographic thalamic inputs directly from the MGv (Fig. 13). In rats, the ventral auditory field, which is frequency organized and placed ventral to the AAF and AI, also receives dense thalamic projections from the caudal part of the MGv (Storace et al. 2010). These findings suggest that the belt regions in rodents receive frequency-organized, lemniscal-thalamic information directly. In contrast, the belt fields with a frequency organization, including the CM, receive thalamic inputs from the frequency-unorganized MGd in marmosets (de la Mothe et al. 2012), suggesting that the frequency representation in the belt may derive from the core after intracortical interactions in primates. Although these present and prior observations suggest that rodents and primates have multiple frequency-organized belt auditory fields, it remains to be seen to what degree these fields are functionally homologous or analogous between rodents and primates.

Many animal species use ultrasonic frequencies for navigation or communication with others. Actually, rodents communicate with each other using ultrasonic frequency vocalization over 50 kHz. Ultrasonic songs produced by male mice induce exploratory behaviors in female mice (Hammerschmidt et al. 2009; Holy and Guo 2005), and isolated pups attempt to communicate with their mother by producing isolation calls (Ehret 2005; Hahn and Schanz 2005). Therefore, it is critical for mice to receive and process ultrasonic sounds. The DM has fewer neurons with low BFs and responds well to ultrasonic sounds compared with the AI (Figs. 5, *D* and *E*, and 6). Moreover, strong plasticity is not induced in the DM by acoustic exposure (Fig. 9). The degree of variability in the frequency organization in cellular resolution recording is less marked in the DM than in the AI (Fig. 6). These data imply that the DM is not dynamically regulated by learning; instead, the DM is dedicated for processing some congenitally important sounds, such as courtship chirps, as shown in Fig. 14.

The DM may play a role of recipient and relay point for processing of courtship songs. The thalamocortical auditory pathway comprises parallel pathways, and each region in the auditory cortex receives a topological projection from the corresponding subregion in the MGv (Horie et al. 2013; Takemoto et al. 2014). The DM receives dense projections directly from the rostral part of the MGv (Fig. 13) and is the first region to be activated in both sexes by a male courtship song. These data imply that the MGv determines the region of the auditory cortex to which song information will be sent. A characteristic waveform and frequency included in voices emitted under a specific condition (Hammerschmidt et al. 2009; Holy and Guo 2005; Insel et al. 1986) may selectively activate gating channels in the thalamus (Schiff et al. 2013) to choose a specific recipient in the auditory cortex. Furthermore, there was a significant difference in response amplitudes of a male courtship song in the DM between sexes (Fig. 14*F*), which may reflect the results of previous behavioral studies that female mice respond specifically to male courtship songs (Hammerschmidt et al. 2009). The auditory cortex is required for fear conditioning using complex sounds (Letzkus et al. 2011). Therefore, subregions of the auditory cortex may themselves have roles for evaluating the biological meanings attached to natural sounds.

## GRANTS

Support for this study was provided by the Japan Society for the Promotion of Science KAKENHI (Grant Number 24700320; to H. Tsukano).

## DISCLOSURES

The authors declare no competing interests.

## AUTHOR CONTRIBUTIONS

Author contributions: H. Tsukano, R.H., M.K., K.T., H. Takebayashi, and K.S. conception and design of research; H. Tsukano, M.H., T.B., and A.U. performed experiments; H. Tsukano, M.H., T.B., and A.U. analyzed data; H. Tsukano, M.H., and K.S. interpreted results of experiments; H. Tsukano prepared figures; H. Tsukano drafted manuscript; H. Tsukano, M.K., H. Takebayashi, and K.S. edited and revised manuscript; H. Tsukano, M.H., T.B., R.H., M.K., K.T., H. Takebayashi, and K.S. approved final version of manuscript.
